# 2469. Comparative Genomics Uncovers Strain Diversity and Possible Unrecognized Transmission of MDROs among Hospitalized Patients

**DOI:** 10.1093/ofid/ofad500.2087

**Published:** 2023-11-27

**Authors:** Dylan Koundakjian, Amanda F Strudwick, Alex Page, Julia Van Riel, D’Ante Gooden, Sarah W Satola, Andrei Bombin, Timothy D Read, Jesse T Jacob, Ahmed Babiker, Michael H Woodworth

**Affiliations:** Emory University Department of Internal Medicine, Atlanta, Georgia; Emory University School of Medicine, Atlanta, Georgia; Emory University School of Medicine, Atlanta, Georgia; Emory University School of Medicine, Atlanta, Georgia; Emory University School of Medicine, Atlanta, Georgia; Emory University School of Medicine, Division of Infectious Diseases, Atlanta, Georgia; Emory University School of Medicine, Atlanta, Georgia; Emory University School of Medicine, Atlanta, Georgia; Emory University School of Medicine, Atlanta, Georgia; Emory University School of Medicine, Atlanta, Georgia; Emory University, Atlanta, GA

## Abstract

**Background:**

Contamination of the healthcare environment by colonized patients is an important source of multidrug-resistant organism (MDRO) transmission. We aimed to determine MDRO room contamination of patients with MDRO clinical cultures to test diversity of dominant strains in colonizing isolates and detect possible transmission.

**Methods:**

Inpatient cases with clinical cultures positive for CRE, extended-spectrum cephalosporin resistant Enterobacterales (ESCRE), CR-Pseudomonas and VRE were eligible for inclusion. Patients on the same unit without positive MDRO clinical cultures in the prior 90 days were selected as controls. Participant inguinal and perirectal sites were sampled by E-swab. Three patient room surface composites were sampled with sponge sticks on the same day. Colonies on differential and selective media were identified by MALDI-TOF with Vitek2 antimicrobial susceptibility testing. Isolates underwent genome sequencing. Clustered gene alignment and average nucleotide identity were compared for conspecific isolates. Transmission was defined as isolation of conspecific MDRO strains with > 99.9% ANI in two or more patients.

**Results:**

Twelve cases and 5 controls were included. Among cases, the same clinical MDRO was detected in 91% (10/11, one patient declined sample collection) of patient-site cultures and in 42% (5/12) of environmental cultures. Among controls, an MDRO was detected in 60% (3/5) of patient samples but no MDROs were found in environmental samples. Antibiotic susceptibility was similar between conspecific patient clinical, colonizing, and environmental isolates. Among isolates sequenced, SNP analysis confirmed genetic relatedness between colonizing and invasive strains and revealed unrecognized unit-based MDRO transmission.

**Figure:**
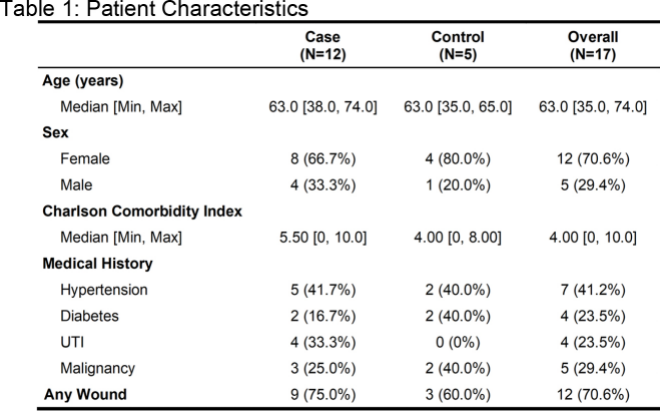

Cases and Controls had similar clinical features

**Figure:**
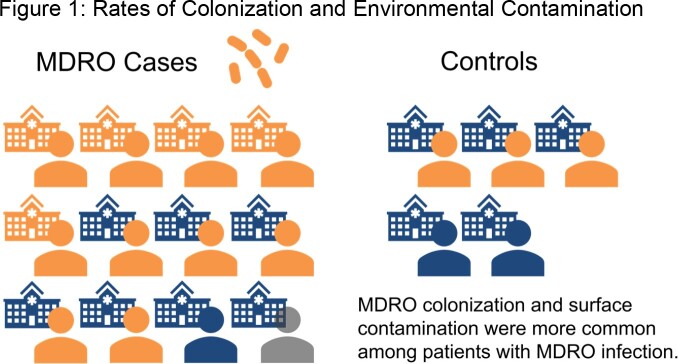

**Figure:**
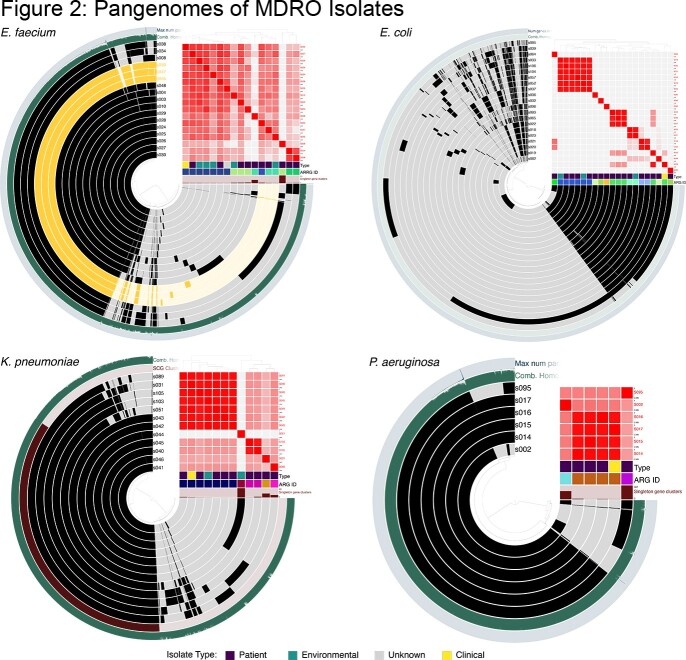

Whole genome sequencing demonstrated patient colonization with multiple conspecific strains and uncovered potential unit-based transmission of colonizing isolates

**Conclusion:**

There is significant environmental contamination from hospitalized patients with clinical MDRO culture positivity. Whole genome sequencing demonstrated patient colonization with multiple conspecific strains and uncovered potential unit-based transmission of colonizing isolates, which may be potentiated by environmental contamination. Further research is needed to understand if reducing patient MDRO colonization could reduce environmental contamination and transmission.

**Disclosures:**

**Ahmed Babiker,** MBBS, Roche: Advisor/Consultant

